# Mitochondrial Oxygenation During Cardiopulmonary Bypass: A Pilot Study

**DOI:** 10.3389/fmed.2022.785734

**Published:** 2022-07-18

**Authors:** Floor A. Harms, Rinse Ubbink, Calvin J. de Wijs, Max P. Ligtenberg, Maarten ter Horst, Egbert G. Mik

**Affiliations:** Department of Anesthesiology, Erasmus Medical Center, Erasmus University Rotterdam, Rotterdam, Netherlands

**Keywords:** mitochondria, mitochondrial oxygenation, cardiopulmonary bypass and maze procedure, acute kidney injury, microcirculation, ischemia

## Abstract

**Objective:**

Adequate oxygenation is essential for the preservation of organ function during cardiac surgery and cardiopulmonary bypass (CPB). Both hypoxia and hyperoxia result in undesired outcomes, and a narrow window for optimal oxygenation exists. Current perioperative monitoring techniques are not always sufficient to monitor adequate oxygenation. The non-invasive COMET^®^ monitor could be a tool to monitor oxygenation by measuring the cutaneous mitochondrial oxygen tension (mitoPO_2_). This pilot study examines the feasibility of cutaneous mitoPO_2_ measurements during cardiothoracic procedures. Cutaneous mitoPO_2_ will be compared to tissue oxygenation (StO_2_) as measured by near-infrared spectroscopy.

**Design and Method:**

This single-center observational study examined 41 cardiac surgery patients requiring CPB. Preoperatively, patients received a 5-aminolevulinic acid plaster on the upper arm to enable mitoPO_2_ measurements. After induction of anesthesia, both cutaneous mitoPO_2_ and StO_2_ were measured throughout the procedure. The patients were observed until discharge for the development of acute kidney insufficiency (AKI).

**Results:**

Cutaneous mitoPO_2_ was successfully measured in all patients and was 63.5 [40.0–74.8] mmHg at the surgery start and decreased significantly (*p* < 0.01) to 36.4 [18.4–56.0] mmHg by the end of the CPB run. StO_2_ at the surgery start was 80.5 [76.8–84.3]% and did not change significantly. Cross-clamping of the aorta and the switch to non-pulsatile flow resulted in a median cutaneous mitoPO_2_ decrease of 7 mmHg (*p* < 0.01). The cessation of the aortic cross-clamping period resulted in an increase of 4 mmHg (*p* < 0.01). Totally, four patients developed AKI and had a lower preoperative eGFR of 52 vs. 81 ml/min in the non-AKI group. The AKI group spent 32% of the operation time with a cutaneous mitoPO_2_ value under 20 mmHg as compared to 8% in the non-AKI group.

**Conclusion:**

This pilot study illustrated the feasibility of measuring cutaneous mitoPO_2_ using the COMET^®^ monitor during cardiothoracic procedures. Moreover, in contrast to StO_2_, mitoPO_2_ decreased significantly with the increasing CPB run time. Cutaneous mitoPO_2_ also significantly decreased during the aortic cross-clamping period and increased upon the release of the clamp, but StO_2_ did not. This emphasized the sensitivity of cutaneous mitoPO_2_ to detect circulatory and microvascular changes.

## Introduction

The use of cardiopulmonary bypass (CPB) during cardiac surgery has a profound effect on the human body since circulation, respiration, homeostasis, and thermoregulation are all affected by the CPB. Maintaining an adequate tissue oxygen supply is essential to preserve cellular oxygenation and subsequently the preservation of organ function during CPB. The supply of oxygen is dependent on the red blood cell (RBC) count, as well as the macrovascular and microvascular perfusion. Inadequate macro-/micro-vascular perfusion and/or shortage of RBCs (anemia) can lead to an inadequate supply of oxygen, resulting in tissue hypoxia. It is widely understood that hypoxia is an independent risk factor for postoperative complications, such as acute kidney injury (AKI), postoperative cognitive dysfunction (POCD), and postoperative delirium (1, 2). Cardiac surgery-associated kidney injury (CSA-AKI) is unfortunately common as the kidneys are especially susceptible to periods of low mean arterial blood pressure and hypoxia due to their high metabolic activity (3). Currently, no individual estimate can determine the minimum mean arterial blood pressure or critical oxygen tension before tissue injury or ischemia occurs. It is known that there is an oxygen reserve that must be depleted before the onset of ischemia. Thus, only a longer period of low oxygen levels contributes to potential ischemia, resulting in acute kidney injury.

On the other hand, striving for a hyperoxic state during CPB has not proven clinically beneficial (4). This is due to the fact that hyperoxia can lead to the generation of oxygen radicals, which play a role in the development of ischemic reperfusion injury (4). Moreover, hyperoxia can lead to alveolar collapse, resulting in atelectasis, which is one of the main contributing factors to postoperative hypoxia (4). This highlights the fact that both hypoxia and hyperoxia can result in undesired outcomes, and thus, a narrow window for optimal oxygenation exists during CPB.

The current perioperative monitoring techniques seem to be sufficient for the maintenance of adequate tissue oxygenation in most patients. However, in high-risk patients, the optimal oxygenation window during CPB is narrow and not completely understood (5). This accentuates the need for novel monitoring techniques that can measure tissue oxygenation. The recently introduced COMET^®^ monitor (Photonics Healthcare, Utrecht) could be of significance for these patients.

The Cellular Oxygen METabolism (COMET^®^) monitor enables non-invasive, *in vivo* measurements of cutaneous mitochondrial oxygenation (mitoPO_2_). In order to measure cutaneous mitoPO_2_, the COMET^®^ device utilizes the protoporphyrin IX–triplet state lifetime technique (PpIX-TSLT) (6–8). This technique relies on the accumulation of an oxygen-sensitive delayed fluorescent porphyrin, namely, protoporphyrin IX (PpIX), which is endogenously generated in the mitochondria through the administration of 5-aminolevulinic acid (6–8). Subsequent PpIX singlet excited-state intersystem crossing into triplet states results in long-lived triplet states that are quenched efficiently by molecular oxygen, which is the only known quencher *in vivo* (6–8). The pioneering work conducted by E.G. Mik validated that the delayed fluorescence quenching by molecular oxygen was able to consistently quantify mitoPO_2_ in isolated cells, isolated organs, and *in vivo* (6). Moreover, the fact that mitochondria are the end-users of oxygen, mitoPO_2_ reflects the local balance between oxygen supply and oxygen consumption within the cell (6, 7, 9). This technique has also been proven feasible in measuring the cutaneous mitoPO_2_ in healthy volunteers (10–12).

Near-infrared spectroscopy (NIRS) is a technique utilized to monitor tissue oxygenation, especially for the prevention of cerebral hypoxia (13). However, Chan et al. conducted a systematic review of 11 randomly controlled trials and found that current reference values for NIRS are varied and that the information on any association between NIRS measurements and outcomes such as POCD is limited (14). In recent laboratory and clinical studies, cutaneous mitoPO_2_ has shown to be more sensitive in measuring subtle changes in local oxygen supply and demand than the NIRS technique. This sensitivity was demonstrated for the first time in a porcine model. In this experiment, hemodilution in pigs resulted in an earlier decrease in the cutaneous mitoPO_2_ compared to the NIRS tissue oxygenation saturation measurements (StO_2_) (15). Second, a recent pilot study in chronic anemia patients (16) has shown variable individual responses to cutaneous mitoPO_2_ measurements during red blood cell transfusion. This further supports the potential of cutaneous mitoPO_2_ measurements to provide an insight into tissue oxygenation. Therefore, it is assumed that cutaneous mitoPO_2_ measurements can be an earlier indicator for tissue hypoxia than StO_2_.

This pilot study's primary aim was to examine the feasibility of this measurement technique during cardiothoracic surgical procedures requiring a CPB. The impact of hemodynamic changes during CPB and its run time on the cutaneous mitoPO_2_ were also analyzed. Examining the impact of CPB run time on cutaneous mitoPO_2_ is essential since it is widely understood that long CPB run times lead to worse clinical outcomes (17). This decrease in clinical outcome is primarily attributed to increased myocardial stunning and increased inflammatory mediators during long CPB run times. The resulting inflammatory reaction contributes to increased vascular permeability, leading to an efflux of intravascular fluid into the interstitial space, causing microvascular dysfunction and an increased distance from the blood circulation to the target cell mitochondria (18, 19). Therefore, the expectation was that long CPB run times are associated with reduced mitochondrial oxygenation, tissue hypoxia, and poor outcomes.

Moreover, the importance or superiority of pulsatile flow during CPB has remained controversial and unresolved (17). There are two major CPB methods, namely, continuous flow or pulsatile flow utilizing a rotary pulsatile flow pump. However, in this academic hospital, only a continuous flow pump is used. Therefore, in order to examine the effects of non-pulsatile flow on a mitochondrial level, cutaneous mitoPO_2_ will be examined during the CPB aortic cross-clamp period (a period in which patients have no pulsatile flow) and will be contrasted to the CPB period in which the patients have their own cardiac output.

In conclusion, we provide an impression of the cutaneous mitoPO_2_ and its behavior during CPB. Cutaneous mitoPO_2_ was also compared to simultaneous StO_2_ measurements. The correlation between perioperative cutaneous mitoPO_2_ measurements and clinical outcomes in cardiac surgery has also been investigated.

## Materials and Methods

### Study Population

This single-center observational pilot study was approved by the local medical ethics committee and was conducted in the Erasmus Medical Center (Erasmus MC), Rotterdam, the Netherlands. The subjects who could participate were patients planned for cardiothoracic surgery requiring CPB with the use of extracorporeal circulation. This included various cardiothoracic operations including a coronary artery bypass graft (CABG), myectomy, cardiac valve replacement or repair, and aortic root replacements.

The inclusion criteria were over 18 years of age, acceptable proficiency in the Dutch language, and cardiac surgery requiring CPB. The exclusion criteria were not having an indication for invasive intra-arterial blood pressure monitoring, the presence of mitochondrial disease, pregnancy or lactation, emergency surgery, intracardiac shunts, or skin lesions on the upper arm or shoulder that could impede cutaneous mitoPO_2_ measurements. Patients eligible and willing to participate signed informed consent forms prior to their surgery.

Patient demographic characteristics, including age, sex, height, and weight, were extracted from their electronic patient files. Intraoperative parameters and laboratory results were automatically recorded using an electronic recording system, such as heart rate, arterial blood pressure, and CPB settings.

### Sample Size

This pilot study is the first observational cutaneous mitoPO_2_ COMET^®^ study in cardiothoracic surgery patients. The effects of variables such as hemodynamic changes caused by CPB and vaso-active agents, on the mitochondrial function were unknown. Therefore, it is difficult to predict the variation in cutaneous mitoPO_2_.

The sample size is thus based on the available data from a study in which the feasibility of cutaneous mitoPO_2_ measurements was examined in healthy volunteers (10). The healthy volunteers had a mean cutaneous mitoPO_2_ of 44 ± 17 mmHg. In oxygen-supplemented patients, cutaneous mitoPO_2_ is expected to be slightly higher and is assumed to be around 55 mmHg.

Therefore, if an accuracy of 10% is assumed with the expected mean 55 mmHg, there is a 5.5 mmHg margin of error. A confidence interval of 95% corresponding to t = 1.96 and s = 17 mmHg as standard deviation gives a sample size n of 36.7 subjects. However, due to the assumptions, an estimation of 37 subjects was made. The fact that this patient population is more heterogeneous than the healthy volunteers results in its own variability, it was determined that a minimum of 41 successfully included subjects was a realistic sample size.

Last, the correct and timely application of the 5-aminolevulinic acid (ALA) plaster is crucial to obtaining an adequate signal quality for reliable cutaneous mitoPO_2_ measurements. To prevent a loss of power, subjects with an initial signal quality (signal quality is calculated and displayed by the COMET^®^ monitor) below 20% were replaced as this indicates a problem in the preparation phase. The cause of failure in cases with low signal quality was examined to ensure proper action for future prevention.

### Cardiopulmonary Bypass

The CPB circuit consists of the Quadrox HMOD 71000 with an integrated Quart HBF140 arterial filter, a venous hard-shell cardiotomy reservoir, and the revolution pump for the heart–lung machine, Stöckert S5 (Sorin Group). All surfaces were coated with Safeline^®^ coating (Maquet Cardiopulmonary) or PHYSIO^®^ coating (Sorin Group). The priming solution consisted of Gelofusine^®^ (B. Braun, Melsungen AG, Germany) and mannitol 200 g/l (Baxter Healthcare, Utrecht, The Netherlands). The initial heparin (Leo Pharmaceuticals, Weesp, The Netherlands) dosage was 300 IU/kg body weight, with an additional 7500 IU in the CPB circuit prime solution. Anti-coagulation through active clotting time was monitored using the Hemochron^®^ Jr. (J.T.C. Europe, Rodano, Italy). Values >440 seconds were considered safe for CPB. The oxygenation was regulated by using in-line blood gas monitoring CDI-500^®^ (Terumo Corporation, Tokyo, Japan) in an α-stat method, combined with a mass flow meter (Brooks Instruments, Hartfield, PA, USA). Intraoperative hemodynamic management targeted an arterial non-pulsatile flow of 2.4 L/min/m^2^ and mean arterial pressure >60 mmHg. The targeted value for PaO_2_ during the CPB time was 150 mmHg. Cardioplegia was induced by the administration of St. Thomas Hospital solution.

### Cutaneous MitoPO_2_ Measurement

Cutaneous mitoPO_2_ measurement is an optical technique based on the PpIX-TSLT (6). The PpIX-TSLT is based on the principle of oxygen-dependent quenching of the excited triplet state of PpIX. The application of the porphyrin precursor ALA induces PpIX in the mitochondria. After photoexcitation with a pulse of green light, PpIX emits delayed fluorescence, the lifetime of which is inversely related to the amount of oxygen (6). The PpIX-TSLT is incorporated in a COMET^®^ clinical monitoring device (7).

The day before surgery, an Alacare plaster (photonamic, Wedel, Germany), containing ALA, was applied on the upper arm or shoulder. Before the induction of anesthesia, the COMET^®^ probe was attached to the pre-medicated skin. The area around the probe was covered with gausses to minimize the interference of ambient light. Cutaneous mitoPO_2_ in mmHg was measured perioperatively with a 5-min interval. At the end of the operation, the COMET^®^ probe was removed, and the skin was covered with a plaster to protect it against light exposure.

The included patients with an initial signal quality below 20% as indicated by COMET^®^ device were excluded and replaced to ensure reliable measurements during the complete surgery period. Cutaneous mitoPO_2_ measurements with an absolute value of 0 mmHg or a signal quality lower than 20%, as reported by the COMET^®^, were excluded.

### StO_2_ vs. Cutaneous MitoPO_2_

StO_2_ was measured using the INVOS^®^ device (Medtronic, Minneapolis, MN), which uses near-infrared spectroscopy to determine the regional oxygen saturation (20). The INVOS^®^ probe was attached to the skin near the COMET^®^ probe. StO_2_ was recorded with a default INVOS^®^ sampling interval of approximately 30 s and was defined as percentage (%) hemoglobin saturation. At the end of the surgery, both the probes of the COMET^®^ and INVOS^®^ were removed simultaneously; the last measurements of both devices were used as a reference to synchronize the two systems in time.

### Start vs. End of CPB Period

There is an expectation that the cutaneous mitoPO_2_ will be negatively impacted by the CPB run time due to previously discussed negative effects. Therefore, the cutaneous mitoPO_2_ values were compared at the start and end of the CPB run time, establishing a baseline with the first 20 min of CPB run time compared to the last 20 min of the CPB period. The StO_2_ measurements were taken during the same time frame as the cutaneous mitoPO_2_ measurements.

### Cutaneous MitoPO_2_ vs. StO_2_ Change Over Time

The cardiothoracic surgical procedures in which CPB is utilized often take around 1 to 3 h. This resulted in the need to cluster the data in order to compare them during operation time; 10-min windows were used to resample both cutaneous mitoPO_2_ and StO_2_ data. For each 10-min time window, a median and interquartile range (IQR) were calculated. For example, if the surgery took 60 min, six time windows were created, and 3 h will correspond with 18 time windows.

### CPB With Aortic Cross-Clamp vs. CPB Without Aortic Cross-Clamp

During cardiothoracic procedures in the Erasmus MC, the heart delivers pulsatile flow and pressure, a pulsatile state. When full flow extracorporeal circulation is given and the aortic cross-clamp is set, a constant flow with a centrifugal pump results in constant blood pressure and flow, a non-pulsatile state. Pulsatile and non-pulsatile periods are both present in one CPB procedure; therefore, the influence on oxygen delivery at the mitochondrial level was also investigated.

CPB without aortic cross-clamp was defined by the presence of cardiac output (arterial blood pressure swings) after the start of extracorporeal circulation till aortic cross-clamping. Even though the CPB pump delivers constant pressure, cardiac output, in addition, creates a pulsatile pressure pattern. When the aorta is cross-clamped and cardioplegia is given by the administration of St. Thomas Hospital solution, the blood flow becomes non-pulsatile defined as CPB with aortic cross-clamp.

### Pulsatile Flow Before and After Aortic Cross-Clamping

To investigate the effect of pulsatile flow on cutaneous mitoPO_2_, two calculation methods were used. In the first method, cutaneous mitoPO_2_ was measured right before and 5 min after aortic cross-clamping. In the second method, cutaneous mitoPO2 was measured before and 5 min after the release of the aortic cross-clamp. This creates two kinds of data within a patient: first, when the aortic cross-clamp is set (pulsatile → non-pulsatile), and second, when it is released (non-pulsatile → pulsatile).

### Pulsatile Flow for Valve Surgery vs. Coronary Bypass Graft Surgery

The effect of pulsatile flow on the cutaneous mitoPO_2_ was compared between patients undergoing valve surgery and CABG. This is due to the fact that after the initiation of CPB, patients undergoing valve surgery receive the aortic cross-clamp almost immediately, while for patients undergoing a CABG procedure, the aortic cross-clamp is placed approximately 30 min later. The reason is that once CPB has commenced during the CABG procedures, the surgeon can properly inspect the coronary arteries and decide where to place the anastomoses. Throughout this time period, the flow is still pulsatile due to the patients' own cardiac output, and only after aortic cross-clamping, the flow becomes non-pulsatile.

**Figure 1:**
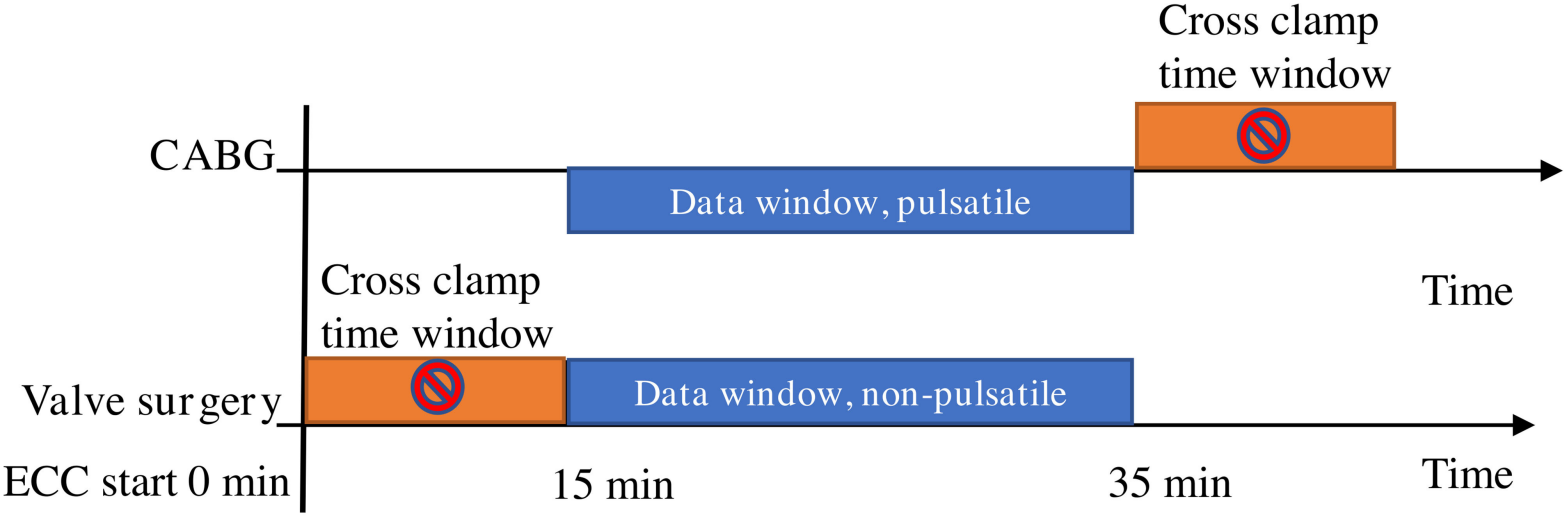
Aortic cross-clamp data window preparation. The window time period is the same for the CABG cohort and the valve surgery cohort, such as 15 min after the start of extracorporeal circulation. In the CABG group, the flow is pulsatile in the data window, and in the valve surgery group, it is non-pulsatile, since the aortic cross-clamp has been set.

After 15 min of extracorporeal circulation during the CPB, the cutaneous mitoPO_2_ data were collected for 20 min. During this time window (15–35 min), the cutaneous mitoPO_2_ was averaged to get a CABG CPB without an aortic cross-clamp data point and a valve surgery aortic cross-clamp data point. This is illustrated in Figure 1, which clarifies the valve surgery with aortic cross-clamp and CABG without aortic cross-clamp groups.

### Time Below a Cutaneous MitoPO_2_ Threshold in Correlation With Acute Kidney Injury

CSA-AKI is unfortunately common and is generally associated with longer CPB run times (3). This is due to a wide range of factors, including hypoxia, as previously discussed in the Introduction. Cutaneous mitoPO_2_ could therefore provide new insight into the prediction of CSA-AKI. In order to investigate this, three different ranges of cutaneous mitoPO_2_ were chosen, namely, cutaneous mitoPO_2_ below 20 mmHg, between 20 and 40 mmHg, and above 40 mmHg. It was hypothesized that the longer the cutaneous mitoPO_2_ is in a low range during CPB, the greater the risk of developing AKI.

The COMET^®^ measurement interval varied occasionally as the interval was changed from every 5 min to every minute, or an extra “single measurement” was taken. To determine the number of minutes below a threshold, the data were interpolated to create a data point for every minute. This was done with an approximation function in R version 3.5.2 (21). The ‘approxfun' in R returns a list of points that linearly interpolate given data points. Having a data point for every minute, the subsequent number of minutes that the cutaneous mitoPO_2_ was within one of the ranges could be determined.

The patients were grouped retrospective in AKI and non-AKI according to the AKI criteria (22).

- An increase in serum creatinine by ≥26.5 umol/L within 48 h; or
- An increase in serum creatinine to ≥1.5 times the baseline, which is known or presumed to have occurred within the prior 7 days; or
- A urine volume <0.5 ml/kg/h for 6 h.

A group comparison was carried out between the AKI and non-AKI groups comparing the absolute number of minutes, and as the percentage of the total CPB run time, the cutaneous mitoPO_2_ was below a threshold.

### Statistics

Normality was tested using the Shapiro–Wilk test. Normally distributed data are presented as means ± SD and non-normally distributed data as medians with 25–75th percentiles. The start and end periods of cutaneous mitoPO_2_, StO_2_, and pulsatility before and after the aortic cross-clamp were compared with a paired Mann–Whitney U-test. Pulsatility for valve surgery and CABG group comparison are done using an unpaired Student's *t*-test.

For categorical data, such as patient characteristics, a two-sided Fisher's exact test was carried out. For continuous values, a Mann–Whitney U test was used. Significance was determined by a *p-*value of <0.05.

## Results

**Figure 2:**
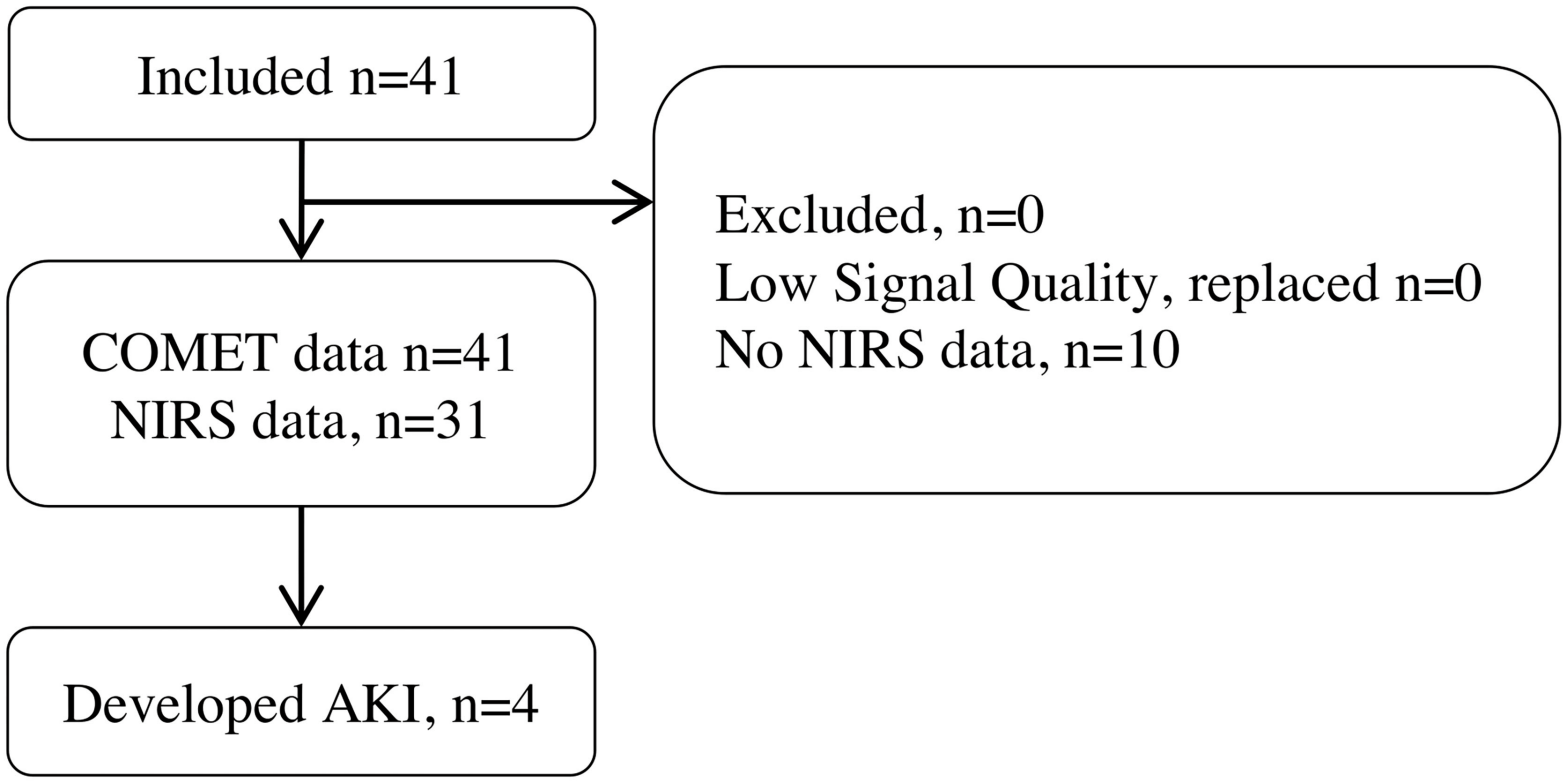
Patient study flowchart.

COMET^®^ data were successfully collected from all 41 patients who underwent cardiothoracic surgery requiring CPB. In total, 0 patient was excluded or replaced, and this is illustrated by a flowchart in Figure 2. The INVOS^®^ device, which was used for StO_2_ measurements, was not available for 10 patients; thus, they do not have any StO_2_ data. This is due to the introduction of a new NIRS monitor which was not used for this study to ensure data compatibility. Table 1 describes the baseline patient characteristics.

**Table 1:** Patient characteristics.

	**All (*n =* 41)**	**Non-AKI (*n =* 37)**	**AKI (*n =* 4)**	***p***
Female (n)	15 (37%)	14	1	1^*^
CABG (n)	13 (32%)	12	1	1^*^
Valve surgery (n)	27 (66%)	24	3	1^*^
Re operation (n)	3 (7%)	3	0	1^*^
Age (years)	65 [57–73]	65 [57–73]	64 [58–70]	1^∞^
Weight (kg)	81 ± 13	81 [71–89]	88 [89– 91]	0.15^∞^
Length (cm)	176 ± 95	174 [170–180]	187 [181–188]	0.17^∞^
Duration surgery (min)	250 [202–297]	250 [201–296]	290 [228–392]	0.33^∞^
Duration CPB (min)	152 [111–200]	148 [106–187]	193 [163–240]	0.13^∞^
Hb preop (mmol/L)	8.35 ± 1.1	8.4 [7.6–9.2]	8.85 [8.30–9.10]	0.65^∞^
Hb begin cpb (mmol/L)	6.0 ± 0.8	5.9 [5.5–6.4]	6.6 [6.0–7.0]	0.34^∞^
Hb end cpb (mmol/L)	5.8 ± 0.8	5.8 [5.2–6.3]	5.6 [5.1–6.0]	0.67^∞^
Delta Creatinine (μmol/L) Post-Pre	−5 [−10–12]	−5 [−11–4]	39.5 [31.5–48.5]	0.001^∞^
eGFR Pre-Op	73 ± 20	81 [61–88]	52 [47–59.8]	0.06^∞^
eGFR Post-Op	75 [50–98]	85 [56–99]	37.5 [32.5–42]	0.004^∞^

* *Fisher's exact test two-sided, ^∞^Mann–Whitney U test represented as mean ± SD or median [IQR]*.

### CPB Start and End Cutaneous MitoPO_2_ and StO_2_ Comparison

The median and IQR of CPB time was 152 [111–200] min. At the start CPB, the median cutaneous mitoPO_2_ was 63.5 [40.0–74.8] mmHg and decreased significantly *p* < 0.01, *n* = 41 to 36.4 [18.4–56.0] mmHg by the end of the CPB time. The average cutaneous mitoPO_2_ was 44.1 [30.6–53.3] mmHg.

**Figure 3:**
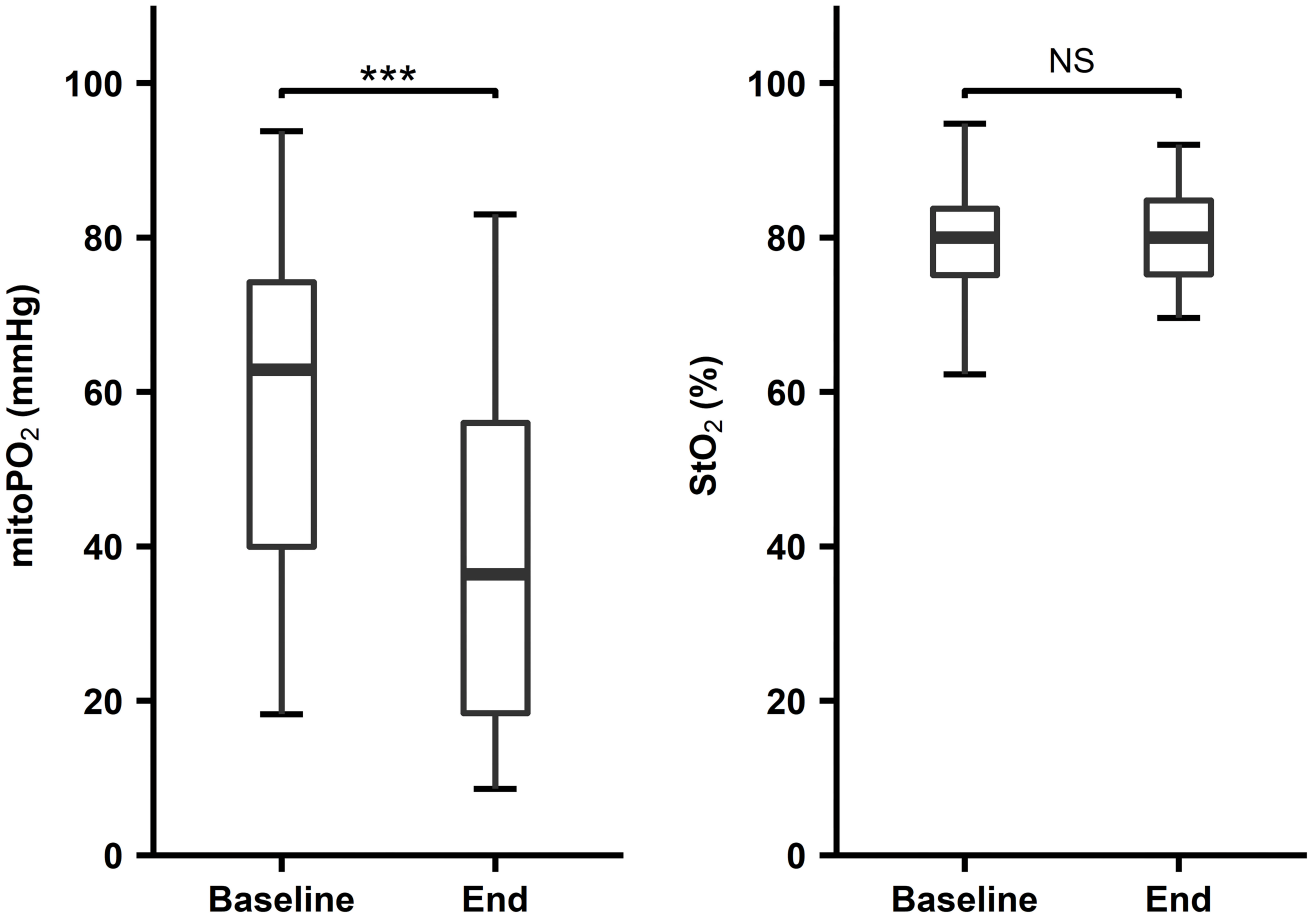
First and last four values during CPB, COMET^®^ cutaneous mitoPO_2_ *n* = 41, ***p < 0.01, INVOS^®^ StO_2_, *n* = 31, *p* = not significant (NS). Boxplot with median, IQR box, and whisker 1.5 times IQR.

The StO_2_ at the start of CPB was 80.5 [76.8–84.3]% and did not change significantly by the end of the CPB time, 80.0 [75.8–84.8]%, *p* = NS, *n* = 31. This is portrayed in Figure 3.

### Cutaneous MitoPO_2_ and StO_2_ Change Over Time

**Figure 4:**
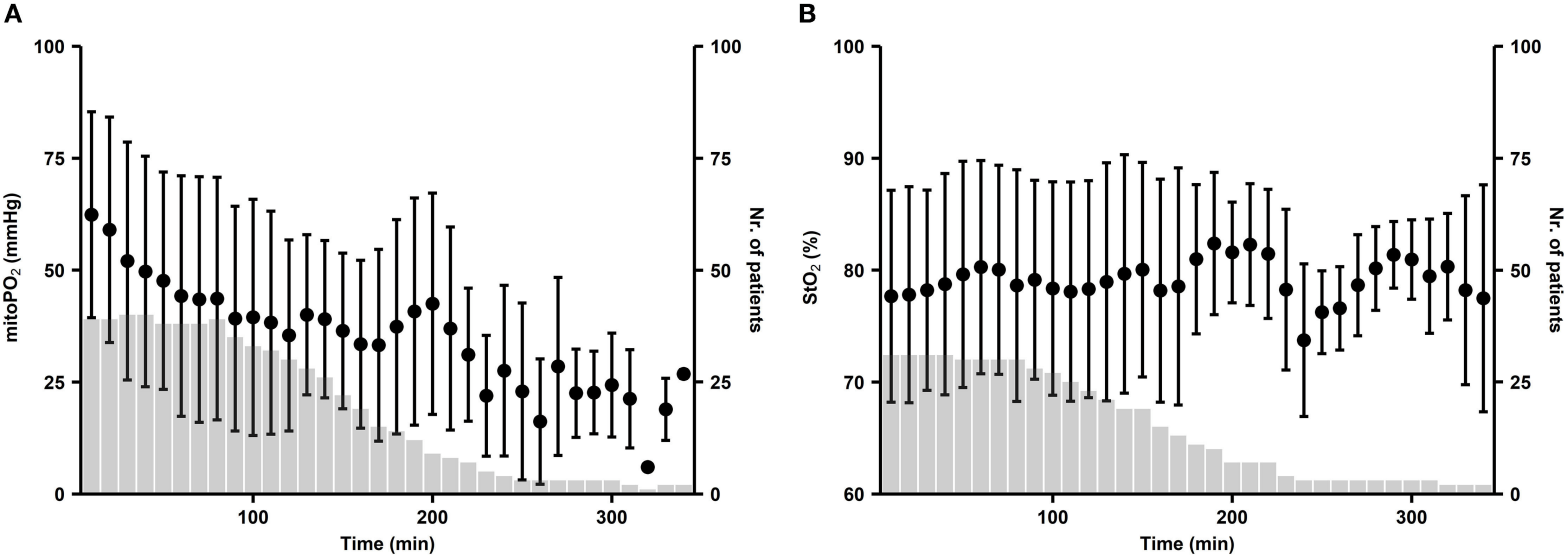
**(A)** Distribution of cutaneous mitoPO_2_ during CPB and **(B)** distribution of StO_2_ during CPB, with the start of CPB at t = 0. Dots represent the mean value of patient-averaged cutaneous mitoPO_2_ per 10-min window. Whiskers demonstrate the standard deviation. Bars represent the number of patients.

For the StO_2_ and cutaneous mitoPO_2_, 10-min data windows from 31 patients were used. Cutaneous mitoPO_2_ started at a mean of 62 ± 23 mmHg and slowly dropped to 44 ± 27 mmHg after 1 h and became steady after approximately 90-min CPB run time (see Figure 4A). Overall, the deviation of cutaneous mitoPO_2_ was large, however, as mentioned in Section CPB Start and End Cutaneous MitoPO_2_ and StO_2_ Comparison. They all had a decrease in the cutaneous mitoPO_2_ during the CPB runs.

A longer duration of CPB run times resulted in lower cutaneous mitoPO_2_ levels. However, StO_2_ showed no clear changes during CPB, as illustrated in Figure 4B.

### Effect of Aortic Cross-Clamping

To determine the effect of aortic cross-clamping on the cutaneous mitoPO_2_, two methods were used. The first method investigated cutaneous mitoPO_2_ pre- and post-aortic cross-clamping.

#### Effect of Aortic Cross-Clamping

**Figure 5:**
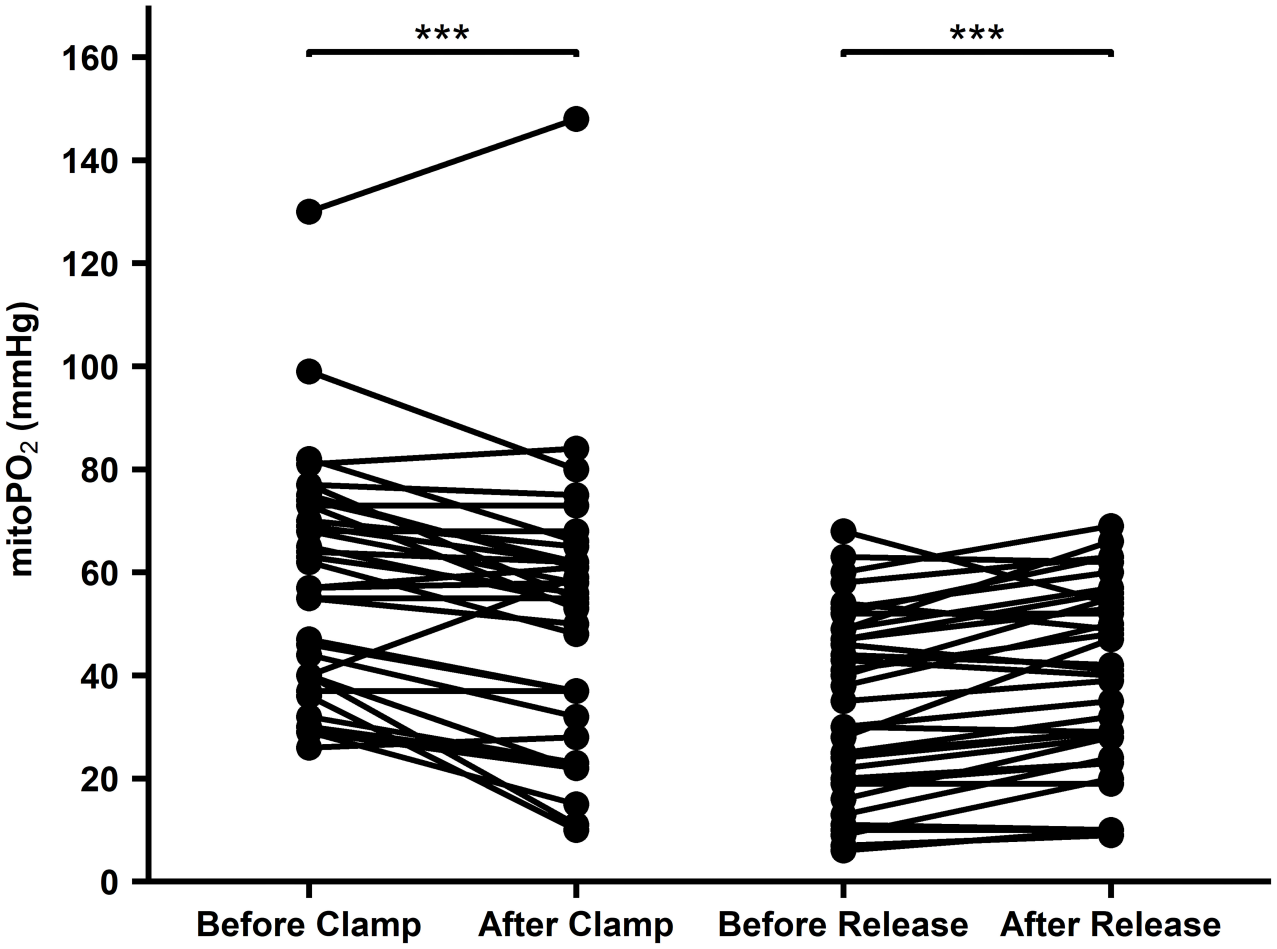
Cutaneous mitoPO_2_ values before and after aortic clamping and cutaneous mitoPO_2_ values before and after the release of aortic clamping. WU test paired *p* < 0.01***.

When the aortic cross-clamp was set, the median cutaneous mitoPO_2_ decreased by 7 mmHg, *n* = 41, *p* < 0.01, and when it was released, it increased 4 mm Hg, *n* = 41, *p* < 0.01, as seen in Figure 5.

#### Effect of Aortic Cross-Clamping at the Start of CPB

**Figure 6:**
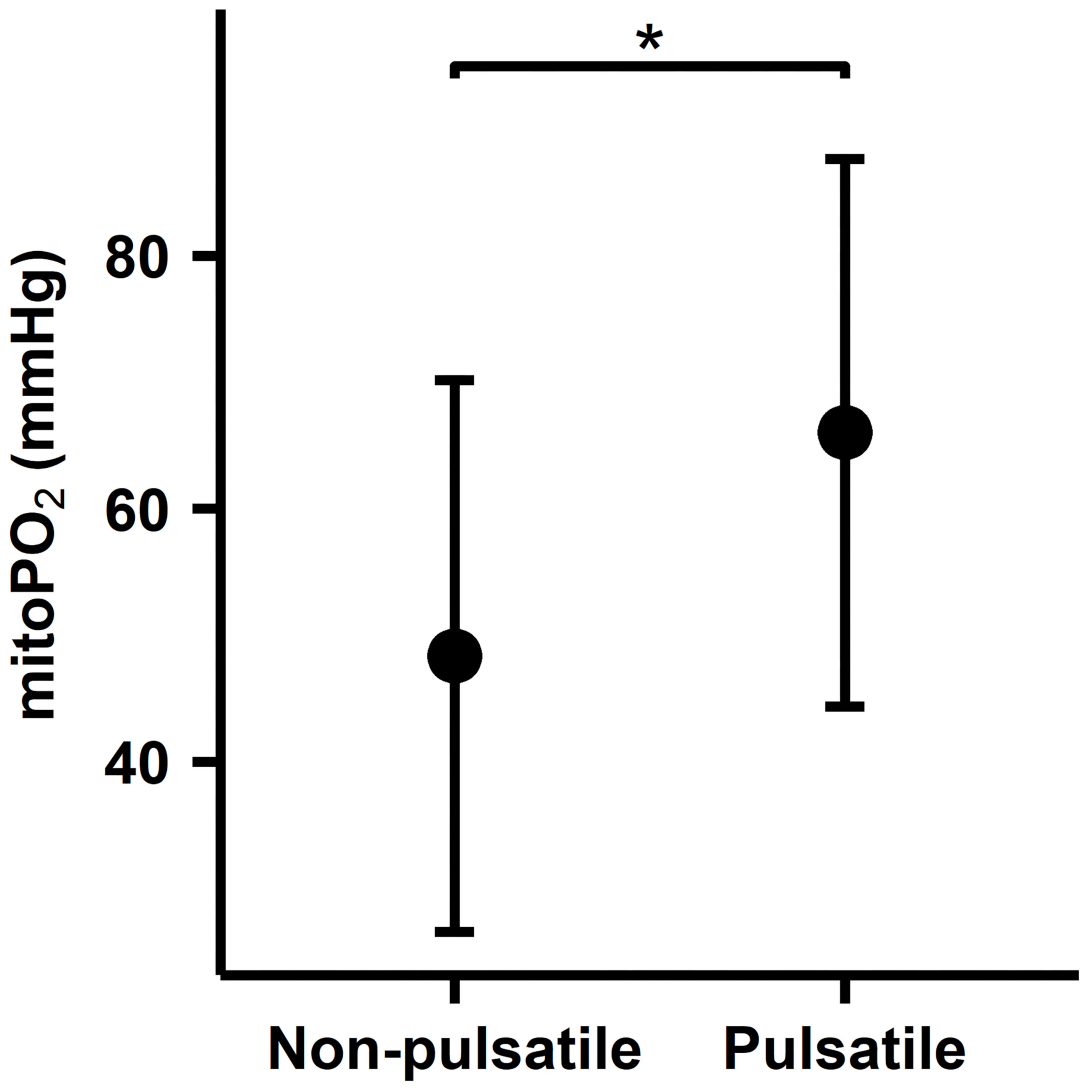
Comparison between valve surgery aortic cross-clamp period and CABG non-aortic cross-clamp period. An average of the 20-min data window after the extracorporeal circulation has been running for 15 min per patient. Mean SD plot of cutaneous mitoPO_2_. *indicates a statistically significant difference *p* < 0.05.

Within the second method, the non-pulsatile flow occurred during the 20-min window after commencing extracorporeal circulation during valve surgery *n* = 19 and was compared to the CABG group *n* = 8, in which the aortic cross-clamp has not been placed. The cutaneous mitoPO_2_ was 17.7 mmHg lower in the valve surgery group than in the CABG group *n* = 8, 48.4 ± 28.8 vs. 66.0 ± 21.7 mm Hg, *p* = 0.03. This is shown in Figure 6.

### Time Below the Cutaneous MitoPO_2_ Threshold in Correlation With Kidney Injury

**Figure 7:**
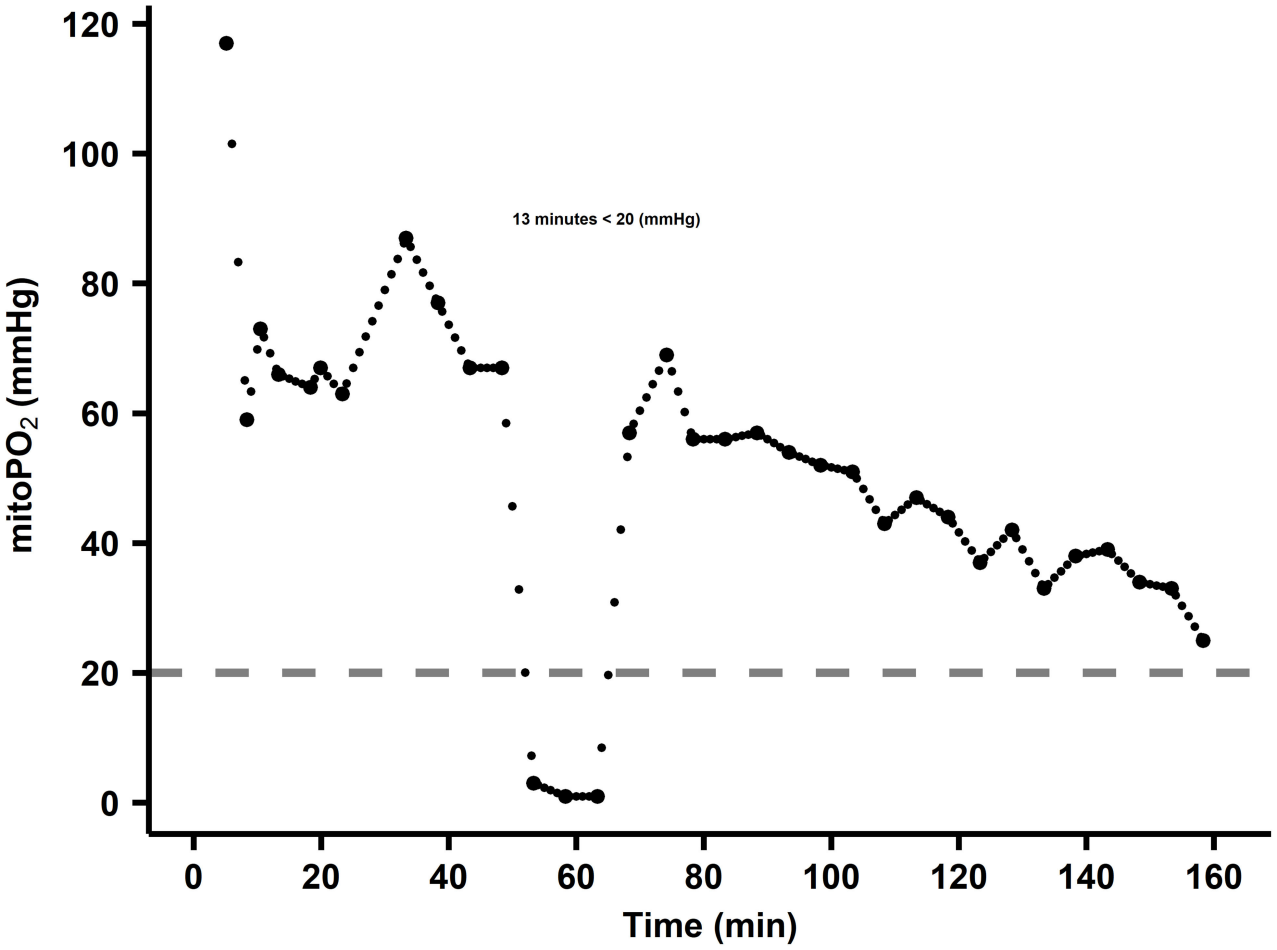
Example of minutes below threshold calculation. The dots are cutaneous mitoPO_2_ values, and the dashed line is the threshold set at 20 mmHg. In this case, the cutaneous mitoPO_2_ was below the threshold for 13 min.

Totally, four of the 41 included patients, 9.8%, developed postoperative CSA-AKI according to the AKICS criteria (22). Figure 7 shows the calculation method that determines the number of minutes the cutaneous mitoPO_2_ was below the set threshold.

**Figure 8:**
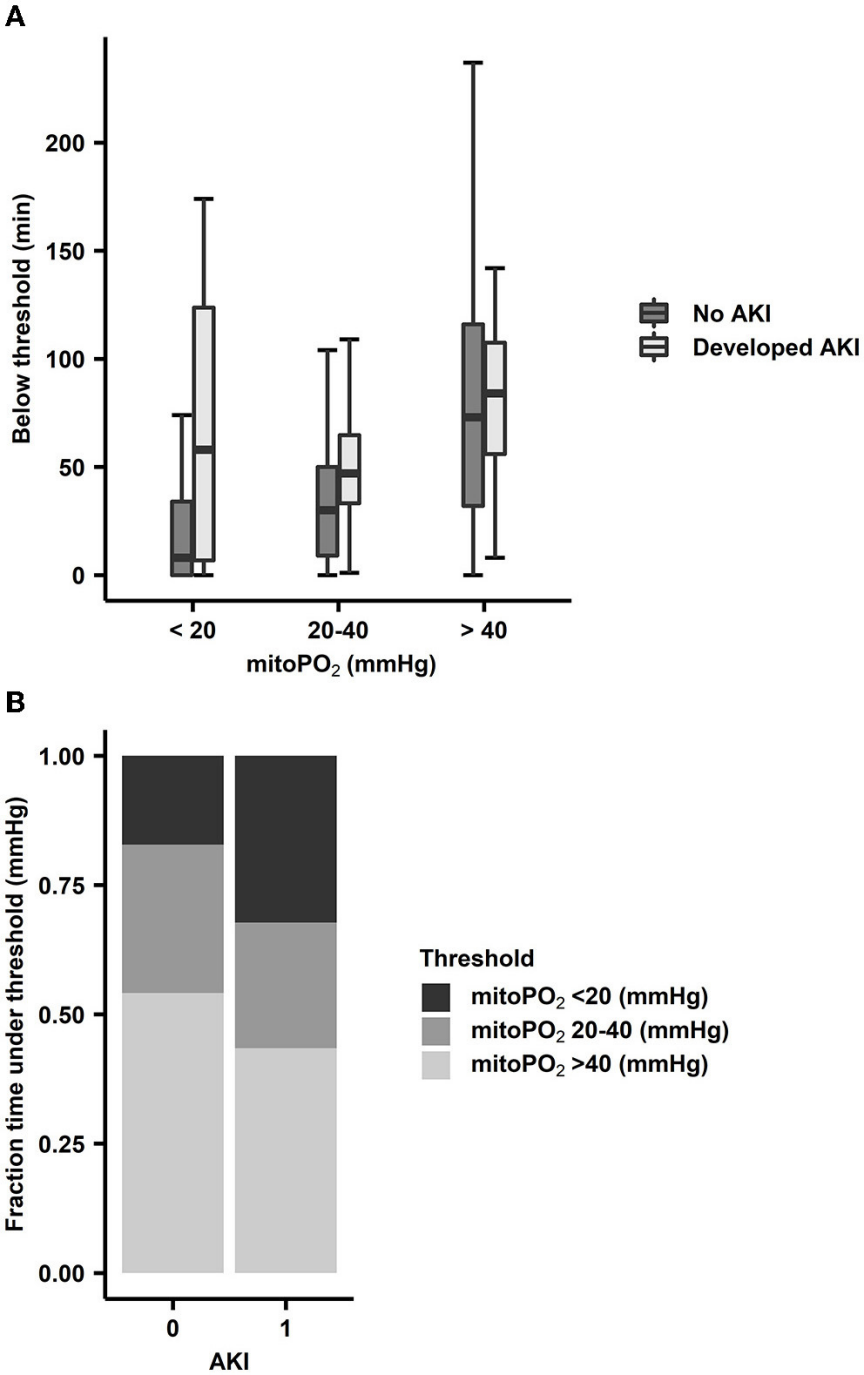
**(A)** Absolute time within cutaneous mitoPO_2_ ranges for AKI (*n* = 4) patients and non-AKI (*n* = 37) patients. The cutaneous mitoPO_2_ ranges were below 20 mmHg, between 20 and 40 mmHg, and above 40 mmHg. Boxplot with median, IQR box, and whisker 1.5 times IQR, and outliers are not shown. **(B)** Time spent within a range as a proportion of CPB/extracorporeal circulation duration. Proportion is achieved by dividing the number of minutes below the threshold by CPB duration.

Within the AKI and non-AKI groups, the number of min below 20 mmHg, between 20 and 40 mmHg, and above 40 mmHg was examined. This resulted in a 50-min median difference in duration in the lowest cutaneous mitoPO_2_ group between AKI (58 min, *n* = 4) and non-AKI (8 min, *n* = 37). The other threshold levels did not show larger differences, as seen in Figure 8A.

When examining the percentage of the procedure time spent below the 20 mmHg threshold, the AKI group spent 32% of the CPB run time below this threshold, whereas the non-AKI group spent 8% of the CPB run time below this threshold, as seen in Figure 8B.

## Discussion

This pilot study demonstrates that it is feasible to examine cutaneous mitoPO_2_ in patients undergoing cardiothoracic surgery requiring a CPB. Cutaneous mitoPO_2_ was successfully measured intraoperatively using the COMET^®^ device based on the PpIX-TLST to monitor oxygen delivery on a mitochondrial level (7). Moreover, it was also evident that it was logistically feasible to measure the cutaneous mitoPO_2_ during cardiothoracic procedures as all 41 patients were measured successfully without any major incidents.

The COMET^®^ enables the non-invasive measurement of *in vivo* cutaneous mitoPO_2_ values. In this study, the mean cutaneous mitoPO_2_ level at the start of surgery was 62 ± 23 mmHg, which is in-line with the typically reported cutaneous mitoPO_2_ levels under baseline circumstances of 40–70 mmHg (9, 23). This is substantially higher than the classically derived and theorized mitoPO_2_ values, which are based on a gradual decline in PO_2_ from the vascular, interstitial, and cytosolic compartments (24–26). Historically, most studies reported low compartmental/tissue PO_2_ levels, with values ranging from 10 to 17 mmHg based on measurements using oxygen electrodes (27, 28). This has resulted in the classically derived and estimated mitoPO_2_ values of several mmHg (24–26). However, after the introduction of less invasive measurement techniques, it was found that tissue oxygen levels were substantially higher than those measured by oxygen electrodes (29). This has been exemplified by Bodmer et al. through the use of phosphorescence-based microvascular PO_2_ measurements of the liver in which they measured tissue oxygen values of 60 mmHg (30), as opposed to classical studies using oxygen electrode measurements in which the measured tissue oxygen was between 10 and 20 mmHg (31). These recent insights into microvascular PO_2_ suggest that the classical estimations of mitoPO_2_ are potentially underestimated and are likely to be in the order of several tens of mmHg (32–34). This is in line with the average cutaneous mitoPO_2_ as measured by the COMET^®^.

It is important to note that cutaneous mitoPO_2_ as measured with the COMET^®^ monitor is an average mitochondrial oxygen tension in the measurement area under the COMET^®^ sensor and not the oxygen tension of a single mitochondrion. The oxygen tension in individual mitochondria is heterogenic because of the presence of an oxygen gradient necessary to drive the oxygen flux. The mitochondria in the measurement site have differing mitoPO_2_ values influenced by the location of each mitochondrion and its oxygen consumption rate. Mitochondria in close proximity to large vessels can have a higher oxygen tension than capillaries, and mitochondria located further away from capillaries and large vessels have a lower mitoPO_2_ (35). Our current understanding of mitoPO_2_ advocates that it should be interpreted as an oxygen balance, depending on both oxygen supply and consumption (36).

The information emphasized in the previous two paragraphs is important for the interpretation and understanding of the cutaneous mitoPO_2_. Until now, there is no clear threshold at which it is safe to say that the cutaneous mitoPO_2_ as measured by the COMET^®^ is too low and at which point an intervention must be made to increase it. In general, our knowledge about *in vivo* mitochondrial oxygen tension and oxygen-dependent functional and metabolic adaptation is limited. *In vitro* studies have previously shown that tissue cells start adapting to available oxygen at higher mitochondrial oxygen levels than isolated mitochondria. Isolated mitochondria only experience oxygen-limited ATP production at very low mitochondrial oxygen levels (<2 mmHg). This is due to the high oxygen affinity of the mitochondrial respiratory chain (37). However, a study examining physiological hearts found that tissue cells started adapting to available oxygenation levels at values as high as 20–40 mmHg (38). This oxygen dependence is driven by oxygen-sensing mechanisms such as the hypoxia-inducible factor (38–40). Such mechanisms result in oxygen-limited ATP production at much higher oxygen levels than previously assumed. However, as mitoPO_2_ is an averaged value of all the mitochondria measured using a sensor, a low mitoPO_2_ value could indicate that some of these mitochondria are hypoxic and in a state of shock while others are not. In order to improve the understanding and interpretation of cutaneous mitoPO_2_, multiple clinical trials are currently being conducted.

NIRS was included in this study as it is a clinically available means to non-invasively assess tissue saturation, a measure for tissue oxygenation but actually measured in the vasculature. Unlike NIRS, in which the tissue saturation values are not calibrated, the values of the cutaneous mitoPO_2_ are calibrated (9). In contrast to the changes exhibited by the cutaneous mitoPO_2_ during the CPB run times, StO_2_ measured by NIRS did not show any significant changes. In our patient cohort, only during extreme intraoperative events such as deep hypothermia with circulatory arrest, StO_2_ decreased. This apparent earlier or more sensitive response of cutaneous mitoPO_2_ to microcirculatory alterations than NIRS has been previously demonstrated in a hemodilution study on pigs. During hemodilution, cutaneous mitoPO_2_ dropped well before StO_2_ did (15).

In this study, we found that cutaneous mitoPO_2_ progressively decreases with the length of the CPB run time. This is further highlighted when comparing the baseline cutaneous mitoPO_2_ at the start of the CPB and the cutaneous mitoPO_2_ at the end of the CPB run, which was significantly reduced. However, after a CPB run time of 180 min, there were few patients left; this potentially resulted in a bias as the differences in cutaneous mitoPO_2_ were especially pronounced after 180 min. As previously mentioned in the Introduction, long CPB run times are associated with poor clinical outcomes (17). This can be due to a myriad of factors, one of which is that patients with prolonged CPB run times are often in a worse preoperative clinical condition requiring more complex surgeries than those with shorter run times augmenting the effect of the CPB run times. However, it is widely understood that a CPB with extracorporeal circulation results in multiple physiological changes such as decreased blood pressure, hemodilution, hypothermia, hyperoxia, and cardiac arrest, and each of these negatively influences the microcirculation (41). Moreover, the heart–lung machine also influences the pharmacokinetics of drugs during CPB run times (42). The heart–lung machine itself negatively impacts the microcirculation as contact of the non-endothelialized synthetic surface with the patient's blood leads to an activation of the innate immune system; this immune reaction associated with CPB carries many similarities to the systemic immune response syndrome (19, 43, 44).

Currently, there is still no consensus on whether pulsatile or non-pulsatile flow is preferred during CPB mainly because no clinical difference in outcome can be found, and there is a lack of clinical monitoring techniques to monitor the differences. This study attempted to evaluate if mitoPO_2_ could detect changes related to pulsatile flow. This was attempted by comparing two distinct surgery groups, namely, the CABG group and the valve surgery group. As previously discussed in the Methods section, these two groups have different states of flow after commencing CPB. Due to the differing aortic cross-clamping times, after cross-clamping, the continuous blood flow is a result of the CPB pump. Cutaneous mitoPO_2_ decreased significantly when the aorta was cross-clamped and increased significantly upon release and re-establishment of partial cardiac output. Other studies demonstrated similar results to Koning et al., showing that the initiation of non-pulsatile flow reduced microcirculatory perfusion and oxygenation (45). Furthermore, when comparing on-pump vs. off-pump surgeries, studies have shown that off-pump CABG surgeries resulted in a less significant decrease in microcirculatory perfusion and oxygenation than on-pump CABG surgeries (41, 46). This can partially be explained by the fact that rhythmic flow variations allow for increased oxygen offloading in the microcirculation (41, 47). These aforementioned studies further reinforce the fact that non-pulsatile flow results in a poor microcirculatory environment, concurring with the cutaneous mitoPO_2_ measurements found in this study.

The changes in cutaneous mitoPO_2_ during aortic cross-clamping and before aortic cross-clamping were not only witnessed on an individual level, as previously mentioned, but also witnessed when comparing patients undergoing CABG and patients undergoing valve surgery. This is because these surgeries have different aortic cross-clamping times after commencing extracorporeal circulation and thus different moments in time when the aorta is cross-clamped. The cutaneous mitoPO_2_ was significantly higher during the period in which cardiac output with extracorporeal circulation co-existed in the 15- to 35-min time frame after commencing CPB in the CABG cohort than during the immediate aortic cross-clamping and CPB flow during valve surgery. This further exemplifies that the patients' own cardiac output most likely improves the oxygen availability in the skin during cardiothoracic surgery requiring a CPB.

The development of AKI is a major postoperative complication after cardiothoracic surgery with an occurrence of approximately 18% (1). The causes of AKI are multifactorial in origin, with no clear single parameter or factor augmentation to prevent AKI. Hypotension and anemia, both of which result in decreased oxygen delivery capacity, will synergistically contribute to the development of AKI (48). However, the cutaneous mitoPO_2_ measured the overall end result of oxygen delivery in which all the factors that potentially contribute to the development in AKI play an important role. Therefore, it is hypothesized that the cutaneous mitoPO_2_ might just be sensitive enough to detect potential perfusion issues. This is mainly due to the fact that poor perfusion in the skin and gastrointestinal tract usually prelude poor perfusion in other organs (49). Cutaneous mitoPO_2_ can be measured as the accumulated time below a certain low oxygen level threshold potentially, highlighting poor perfusion. In this study, a method has been developed to determine the amount of time in minutes below a set threshold of cutaneous mitoPO_2_.

When comparing the duration of time that the cutaneous mitoPO_2_ was below a certain threshold in the AKI and the non-AKI group, an association was found between the duration of time they were below certain thresholds. This is most evident when the cutaneous mitoPO_2_ was below 20 mmHg where the AKI group spent more time below this threshold in absolute minutes and as a percentage of the operation's duration. However, there was a bias in these data as the more prolonged procedures also had generally lower cutaneous mitoPO_2_ values, and the negative effects associated with prolonged CPB run times, such as an increased inflammatory response, also become more pronounced (17). It is also important to note that the eGFR in the AKI group was lower than that in the non-AKI group as a lower eGFR is a risk factor for the development of AKI. Nonetheless, as this study was not powered for an AKI and non-AKI comparison, it is not possible to draw any substantial conclusions, but this is of great interest in further research utilizing the COMET^®^.

In conclusion, this pilot study was successful in demonstrating the feasibility of this measurement technique during cardiothoracic surgical procedures. Cutaneous mitoPO_2_ seems more sensitive to hemodynamic alterations during these surgeries than StO_2_. The COMET^®^ device could potentially become a vital supplementary monitoring technique during cardiothoracic surgeries; however, further specific research must be conducted to prove this.

## Data Availability Statement

The original contributions presented in the study are included in the article/supplementary material, further inquiries can be directed to the corresponding author.

## Ethics Statement

The studies involving human participants were reviewed and approved by Medisch Ethische Toetsings Commissie Erasmus MC. The patients/participants provided their written informed consent to participate in this study.

## Author Contributions

FH and MH conceived and planned the experiments. RU, FH, and ML carried out the experiments. CW, RU, FH, ML, and EM contributed to the interpretation of the results. FH, RU, and CW took the lead in writing the manuscript. All authors provided critical feedback and helped shape the research, analysis, and manuscript. All authors contributed to the article and approved the submitted version.

## Funding

This study was funded by the Young Investigator Grant of the Dutch Society of Anesthesiology and the Department of Anesthesiology in the Erasmus Medical Center, Rotterdam, the Netherlands.

## Conflict of Interest

EM is listed as an inventor on patents related to mitochondrial oxygen measurements held by the Academic Medical Center Amsterdam and the Erasmus Medical Center Rotterdam, the Netherlands. He is the founder and shareholder of Photonics Healthcare, a company that holds exclusive licenses to these patents and that markets the COMET^®^ system. RU is a minority shareholder of Photonics Healthcare. The remaining authors declare that the research was conducted in the absence of any commercial or financial relationships that could be construed as a potential conflict of interest.

## Publisher's Note

All claims expressed in this article are solely those of the authors and do not necessarily represent those of their affiliated organizations, or those of the publisher, the editors and the reviewers. Any product that may be evaluated in this article, or claim that may be made by its manufacturer, is not guaranteed or endorsed by the publisher.

## Abbreviations

AKI: acute kidney injury
ALA: 5-aminolevulinic acid
CABG: coronary artery bypass graft
COMET^®^: Cellular Oxygen METabolism
CPB: cardiopulmonary bypass
CSA-AKI: cardiac surgery-associated acute kidney injury
ECC: extracorporeal circulation
Erasmus MC: Erasmus Medical Center
IQR: interquartile range
mitoPO_2_: mitochondrial oxygen tension
NIRS: near-infrared spectroscopy
POCD: postoperative cognitive dysfunction
PpIX: protoporphyrin IX
PpIX-TSLT: protoporphyrin IX–triplet state lifetime technique
RBC: red blood cell
StO_2_: tissue oxygenation.
